# Sense of coherence in family caregivers of people living with dementia: a mixed-methods psychometric evaluation

**DOI:** 10.1186/s12955-019-1114-0

**Published:** 2019-03-11

**Authors:** Jacki Stansfeld, Martin Orrell, Myrra Vernooij-Dassen, Jennifer Wenborn

**Affiliations:** 1 Division of Psychiatry, University College London, Maple House, 149 Tottenham Court Road, London, W1T 7BN UK; 2 Research and Development, North East London NHS Foundation Trust, Goodmayes Hospital, Ilford, IG3 8XJ UK; 3 Institute of Mental Health, University of Nottingham, Triumph Road, Nottingham, NG7 2TU UK; 4 Radboud University Medical Centre, Postbus 9101, 6500 HB Nijmegen, The Netherlands

**Keywords:** Dementia, Alzheimer’s’, Caregiver, Measurement, Psychometrics, Validity, Reliability, Sense of coherence

## Abstract

**Background:**

Family caregivers of people living with dementia can experience feelings of burden and stress but the concept of sense of coherence has been identified as an important protective trait against the negative impact of caregiving. Despite this, there has been no psychometric evaluation of the Sense of Coherence scale-13 with this population. Therefore, a psychometric evaluation was conducted using a mixed-methods approach.

**Method:**

Five hundred and eighty-three caregivers of people living with dementia participated in the study. We examined the feasibility, internal consistency, construct validity, floor and ceiling effects, concurrent validity and face validity of the Sense of Coherence scale-13.

**Results:**

The Sense of Coherence scale-13 demonstrated adequate internal consistency. Sense of coherence was positively related to resilience, sense of competence and health related quality of life, demonstrating good concurrent validity. However, the face validity of the scale was assessed as poor.

**Conclusion:**

The sense of coherence scale performed well under psychometric evaluation however guidance for caregivers should be examined and revised to reflect feedback from caregivers who completed this study, which could lead to improved face validity for this scale.

**Trial registration number:**

ISRCTN10748953. Registered 18th September 2014.

## Background

By 2050, it is estimated that there will be over 131 million people with dementia worldwide [1], of whom the majority will be cared for by friends or relatives in the community [2]. Over time, family caregivers may experience feelings of burden and stress [3–5]. The negative impact of caregiving can lead to poor outcomes in caregivers, such as depression, anxiety and physical health problems [6, 7]. Protective psychological factors can reduce the negative impact of caregiving. One such protective factor is sense of coherence. The concept of sense of coherence emerged from Antonovsky’s (1979) salutogenic theory [8], which outlines the unique way in which each person views the world and their individual circumstances as comprehensible, manageable and meaningful. This theory focuses on personal strengths as determinants for quality of life and positive wellbeing [9]. Sense of coherence has increasingly been viewed as an important concept within research with family caregivers of people living with dementia. In this population, sense of coherence has been shown to predict health related quality of life [10] and burden [11] and has been related to the ability to cope with the caregiving role [12].

A number of scales have been developed to measure sense of coherence. The original, 29-item sense of coherence scale was reduced to a 13-item measure, the sense of coherence scale-13 items (SOC-13) [9, 13], which is considered a suitable length for research and clinical practice [14]. There are a number of empirical studies incorporating measures of sense of coherence in the dementia family caregiver population (e.g. [11, 15, 16]), however, to our knowledge; there is currently no psychometric evaluation of the sense of coherence scale with family caregivers of people living with dementia. Sense of coherence is important for understanding how to maintain wellbeing and has the potential to be incorporated as an outcome measure in clinical interventions. Therefore, it is important to investigate whether a short sense of coherence measure can be a valid and useful measure for this population.

### Aim

The aim of this research was to examine the psychometric properties (feasibility, subscales, internal consistency, floor and ceiling effects, construct validity and face validity) of the SOC-13 in a population of family caregivers of people with dementia.

## Method

### Design

This was a cross-sectional, mixed-methods study. A mixed-methods design was chosen in order to examine both the psychometric properties of the scale but the caregiver’s experience of completing the scale. The quantitative enquiry can therefore be combined with the rich insights of the qualitative data. All participants were recruited within a period of 6 months. Respondents were contacted through either the Join Dementia Research (JDR) database, an online database of people with dementia and their family caregivers who have registered their interest in taking part in UK-based research or relevant charitable organisations for older people, people with dementia and their family caregivers. All participants were asked to read an information sheet before participating and gave informed consent to take part in the study.

### Participants

Caregivers who expressed an interest in the study following advertisements or mail-outs through the JDR or charitable organisations were emailed or contacted by phone with a link to the online version of the survey and offered a paper copy with a freepost envelope for them to return the completed survey if they preferred. To participate in the research, participants had to be aged 18 or over, currently caring for a person living with dementia in the community and able to read and communicate in English. Participants were excluded from the study if the person with dementia they were caring for lived in residential care.

### Measures

There were two sections to the survey. The first asked demographic questions about the family caregiver and the person with dementia, such as gender, age, living situation, ethnicity and marital status. The second section included standardised measures as outlined below.

#### Sense of coherence

The Sense of Coherence scale-13 [13] is a 13-item scale adapted from a longer 29-item scale [9]. This scale consists of three subscales: meaningfulness (four items), comprehensibility (five items) and manageability (four items). Each item contains 7 response options, which change between questions. Five variables (items 1, 2, 3, 7 and 10) are negatively worded and are therefore reverse coded when summing the items to reach an item total. A higher score represents a higher sense of coherence. Reliability estimates for this scale range from α = 0.70–0.92 [14].

#### Resilience

The Resilience Scale-14 (RS-14) [17] is a 14-item scale used to measure levels of resilience. Items are scored between 1 (strongly disagree) and 6 (strongly agree), with possible total scores ranging from 14 to 84. The higher the score, the higher the level of resilience. The RS-14 has demonstrated good levels of reliability with Cronbach’s α scores ranging between 0.82 and 0.94 [18, 19].

#### Sense of competence

The Short Sense of Competence Questionnaire (SSCQ) [20] is a 7-item measure derived from a longer, 27-item Sense of Competence measure. Scores can range from 7 to 35 and items are scored on a 5-point Likert scale from 1 (agree very strongly) to 5 (disagree very strongly), with a higher score indicating a higher sense of competence. The reliability for the scale is good, with Cronbach’s α scores at 0.76 [20].

#### Self-efficacy

The Self-efficacy for Managing Dementia Scale (SEMD) [21] is a 10-item scale that measures the level of self-efficacy caregivers experience for managing the task of caregiving. Possible scores range from 10 to 100, with higher scores indicating higher self-efficacy. Responses are arranged on a likert scale from 1 (not at all certain) to 10 (very certain). The reliability for this scale is good (α = 0.77) [21].

#### Health related quality of life

The EQ-5D-5 L [20] provides a simple descriptive profile and a single index value for health status, which can be used to evaluate quality of life. Higher scores indicate higher health related quality of life. Cronbach’s alpha has been reported at 0.85 [22].

### Face validity

Five open text-box questions were included directly after the respondents completed the sense of coherence scale, in order to assess face validity. These questions were preceded by an explanation of the sense of coherence scale and what the scale intended to measure and were as follows:

1. Did you find any of the 13 questions difficult to understand?
2. Is there anything you would add to the 13 questions above?
3. Is there anything you would change in the 13 questions above?
4. Is there anything you would remove from the 13 questions above?
5. Do you have any other comments about the 13 questions above?

## Data analysis

Data collected using the online SurveyMonkey format was downloaded from to the IBM SPSS Statistics version 25 [23] and entered manually from the paper surveys. Data was screened for any inconsistencies or errors in data entry. Qualitative data were downloaded or entered separately onto an excel spreadsheet and analysed using inductive thematic analysis [24] by two researchers (JS & JW). The quantitative and the qualitative data was then utilised in tandem to understand the psychometric properties and acceptability of the scale to caregivers.

The feasibility of the scale was calculated by examining the response rate of the scale and an investigation of missing values per item. Internal consistency was examined using Cronbach’s α, which was calculated for the scale as a whole and for each of the subscales. Cronbach’s α is considered acceptable if it falls between 0.7–0.9.

A confirmatory factor analysis (CFA) was conducted in order to explore whether the SOC-13 adequately measured the three domains (comprehensibility, manageability and meaningfulness) as established in the original measure. Confirmatory factor indices values of >.90, SRMR values of <.08 and RMSEA values of between .06 and .08 were considered acceptable.

Floor and ceiling effects are considered important in psychometric evaluation because if these effects are present, it limits the scale’s ability to detect a change in the construct. Floor and ceiling effects were investigated by generating histograms and calculating frequencies to examine the proportion of scores that fell within the highest and lowest possible scores on the scales. Floor effects were considered to be present if above 15% of respondents had the lowest possible scores. Ceiling effects were considered to be present if 15% of respondents had the highest possible scores on the scale as in previous psychometric research [25].

Concurrent validity was calculated using Pearson’s correlation coefficients to examine the relationship between sense of coherence and the other measures, based on previous research and an underlying theory of sense of coherence. Relationships between the variables were considered low if r < 0.29, moderate if r < 0.3 to r < 0.40 and high if r < 0.41 to r < 1 (Field, 2009) [26]. Hypotheses in terms of the relationships between variables were specified a priori:

1. Sense of coherence will be positively correlated with caregiver’s resilience. It is plausible that the way in which a person views their life may impact their resilience to deal with stressful or difficult circumstances. This association has been found in previous literature [27, 28].
2. Sense of coherence will be positively correlated with sense of competence and self-efficacy for managing dementia, as theoretically, an ability to find meaning and understanding in the caregiving situation (sense of coherence) could lead to a higher perceived level of competence and higher self-efficacy to deal with the task of caregiving. Self-efficacy and sense of competence were both assessed in relation to sense of coherence as despite a conceptual overlap, they measure distinct concepts.
3. Sense of coherence will be positively associated with health-related quality of life. This relationship has been demonstrated in previous literature [10].

## Results

583 caregivers of people living with dementia completed the survey. Of these, 516 (89%) participants opted to complete it online and 67 (11%) of participants completed a paper version. There were no significant differences between responses on the online and paper questionnaire in terms of skewness and kurtosis of the scales, scores on the standardised measures and most of the key demographics. There was however, a difference between the ages of family caregivers who completed the online and paper questionnaire (t = − 6.7, *p* = .00). Caregivers who completed the online questionnaire were significantly younger (mean age = 58.3) than those who completed the paper version (mean age = 69).

### Demographics of the family caregivers and people with dementia

The mean age of caregivers taking part in the survey was 59.5 years, with a range of 18–89 years. The majority of family caregivers were female (80.3%), white British or Irish (94.2%) and adult child caregivers (58.9%). Over half of the caregivers were married (68.9%), with 71.3% having completed further education. The people with dementia that were cared for had a mean age of 80 years old, with a range of 39–99. The majority were female (63.4%) and half were married (50.3%). Almost all were white British or Irish (95.2%) and the majority had completed secondary education (55.2%). Half of the people with dementia had an Alzheimer’s disease diagnosis (50.5%). Demographics of the family caregivers and people with dementia is further detailed in Table 1. Where totals do not add to 583, it indicates that some carers did not leave responses for the demographic questions.

**Table 1:** Family caregiver and person with dementia descriptive demographics in the whole sample (*n* = 583)

Family caregiver demographics	Total
Gender *n* (%)	
Female	468 (80.3)
Male	114 (19.7)
Age *M (SD)*	59.5 (12.3)
Range	18–89
Marital status *n* (%)	
Single	81 (14.1)
Married	397 (68.9)
Separated	13 (2.3)
Divorced	42 (7.3)
Widow/widower	13 (2.3)
Other	28 (4.9)
Prefer not to say	2 (0.3)
Ethnicity *n* (%)	
White British/ Irish	543 (94.2)
Mixed British	4 (0.7)
Indian/British Indian	4 (0.7)
Black Caribbean/African	4 (0.7)
Other	19 (3.7)
Highest completed level of education *n* (%)	
Primary education or less	3 (0.5)
Secondary education	136 (23.7)
Further education	409 (71.3)
Other general education	20 (3.5)
Prefer not to say	6 (1)
Relationship to person with Dementia *n* (%)	
Spouse/partner	173 (30.1)
Son/daughter	338 (58.9)
Other	63 (11)
Cohabiting *n* (%)	
Yes	245 (42.7)
No	329 (57.3)
Receiving additional support n (%) (*n* = 572)	
Yes	390 (68.2)
No	182 (31.8)
Person with dementia demographics	
Gender *n* (%)	
Female	369 (63.4)
Male	213 (36.6)
Age *M (SD)*	80 (9.5)
Range	39–99
Marital status *n* (%)	
Single	17 (3)
Married	287 (50.3)
Separated	10 (1.8)
Divorced	25 (4.4)
Widow/widower	227 (39.8)
Other	5 (0.9)
Prefer not to say	–
Ethnicity *n* (%)	
White British/ Irish	541 (95.2)
Mixed British	–
Indian/British Indian	5 (0.9)
Black Caribbean/African	5 (0.9)
Other	4 (0.7)
Highest completed level of education *n* (%)	
Primary education or less	30 (5.3)
Secondary education	313 (55.2)
Further education	163 (27.9)
Other general education	28 (4.9)
Prefer not to say/not known	33 (5.9)
Dementia diagnosis *n* (%)	
Alzheimer’s Disease	288 (50.5)
Vascular	108 (18.9)
Dementia with Lewy	19 (3.3)
Bodies	22 (3.9)
Fronto-temporal	133 (23.3)

### The profile of sense of coherence

Generally, the sense of coherence scores had a normal distribution but demonstrated a positive skew in the entire population, indicating the tendency of family caregivers of people living with dementia to report higher levels of sense of coherence. The mean sense of coherence score was 60.2 and the standard deviation of scores was 14. There was a total range from 17 to 90 across this population.

### Feasibility

Some participants opted out of completing the full survey and therefore there was a completed sense of coherence scale received from 547 family caregivers of people living with dementia. However, there were no missing values or items in any of the returned sense of coherence scales, indicating good feasibility in the measure itself.

### Internal consistency

Overall, the SOC-13 demonstrated good internal consistency (α = 0.883). The comprehensibility subscale had good internal consistency (α = 0.76) and the range of item-total correlations ranged from r = 0.176 to 0.608. In the manageability subscale, Cronbach’s α was good, at α = 0.705 and the range of item-total correlations ranged from r = 0.207–0.667. In the meaningfulness subscale there was good internal consistency (α = 0.72) and the range of item-total correlations ranged between r = 0.39–0.63. All of the item-total correlations were > 0.3. The smallest item-total correlation was for item 3 (r = 0.39, *p* < .001).

### Construct validity

A CFA indicated that the items did not confirm the originally proposed three factor structure. Syntax was entered into MPlus to specify the three latent factors, ‘meaningfulness’, ‘manageability’ and ‘comprehensibility’. These latent factors were allowed to correlate freely. The results indicated that the proposed model was not an adequate fit, with indices falling below or above acceptable limits (Table 2). However, all factor loadings were significant and ranged from 0.419–2.124. There was a small amount of covariance between the subscales.

**Table 2:** CFA validation of the original 3 factor structure of the SOC-13

	×2	df	CFI	RMSEA	SRMR
3 Factors	424.529*	62	0.867	0.103	0.056

×2 = Chi-Square goodness of fit; *df* degrees of freedom, *RMSEA* Root Mean Square Error of Approximation, *SRMR* Standardised Square Root Mean Residual. *Statistically significant at p < .001

### Floor and ceiling effects

Floor and ceiling effects were not present in any of the items. None of the participants scored the maximum or minimum score on this scale.

### Concurrent validity

The hypotheses for concurrent validity were confirmed. Sense of coherence was strongly and positively correlated with resilience (r = 0.56, *p* < .001). Sense of coherence was also moderately and positively correlated with sense of competence (r = 0.42, *p* < .001), and self-efficacy for managing dementia (r = 0.46, *p* < .001). These results indicate that as sense of coherence increases, so does resilience, self-efficacy and sense of competence. Sense of coherence was also moderately correlated with health-related quality of life (r = − 0.38, *p* < .001). As health-related quality of life is reverse scored, a negative correlation indicates that as sense of coherence increases, so does health related quality of life (Table 3).

**Table 3:** Pearson’s correlations for sense of coherence

	Sense of competence	Self-efficacy	Health-related quality of life	Sense of coherence
Resilience	.254***	.455***	.301***	.561***
Sense of competence	–	.354***	.275***	.419***
Self-efficacy	–	–	.223***	.457***
Health-related quality of life	–	–	–	−.379***

* *p* < .05, **p < .01, *** < *p* < .001

### Face validity

A total of 462 caregivers gave at least one comment in the open-text boxes provided. Thematic analysis identified the following themes related to the (1) question content, (2) scoring and (3) relevance to caregiver role. Each of the themes is discussed below and illustrated with data excerpts.

#### Question content

Respondents felt that the questions made them think about their situation and this was considered a positive thing, as it gave them a chance to consider their feelings and reflect upon their situation.

*‘Made me realise how competence and capable I am and how much I love my husband at this stage in his life…our lives’ [female, aged 72]*

However, some caregivers reported that the questions were vague and did not give enough detail as to enable an answer or to orientate them.

*‘Some [questions] are ambiguous, some too vague e.g.: “do you have the feeling that you’re being treated unfairly?” By whom? My relative? My family? Society? Life in general?’ [male, aged 57]*

Caregivers noted problems with specific questions, particularly question 10, ‘Many people - even those with a strong character - sometimes feel like sad sacks (losers) in certain situations. How often have you felt this way in the past?’ Generally, caregivers took offence at the word ‘loser’ and did not recognise the term ‘sad sacks’ as this is not a common term used in the UK.

*‘Losers is not a very helpful phrase’ [Male, aged 85]*

In addition, caregivers reported confusion with question 12, “How often do you have the feeling that there’s little meaning in the things you do in daily life?” They felt that meaning was a very ambiguous term and not easy to categorise.

*‘The question about ‘meaning’ is an odd one. I know my life has little meaning in the grander scheme, but that doesn’t mean I’m not happy in my little bit.’ [Female, aged 49]*

#### Scoring

The nature of the scoring created problems for respondents in terms of choosing the difference between scores on the scale and quantifying scores between 1 and 7. As the response options changed with each question, caregivers found this confusing and sometimes difficult to select the most relevant number.

*‘They aren’t easy to quantify which made them tricky to answer’ [male, aged 57]*

#### Relevance to caregiver role

Some of the response options were difficult for the caregivers to answer as they felt that there was no constant when living with someone with dementia. Life was not predictable and may change from one day to the next.

*‘[The questions are] not [difficult] to understand just difficult to answer as there is not a constant in…living with someone with dementia’ [Female, aged 53]*

Dementia is a degenerative condition and therefore caregivers felt that their response would change as their friend/relative’s dementia progressed and became more severe.

*‘This only applies to now, things may change as my husband’s condition deteriorates’ [Female, aged 75]*

Caregivers also felt the instructions for the sense of coherence scale and the questions were not specific enough. Caregivers reported the need for distinction as to whether the questions were aiming to reflect their life in general or their caregiving role specifically in relation to the person with dementia that they support.

*‘I found it hard to disentangle responses in relation to how I feel about coping with and supporting my mum with Alzheimer’s and the other part of my life.’ [Female, aged 61]*

## Discussion

The strength of this study is that it provides a psychometric evaluation using a mixed methods approach, of a well-used and popular scale in a new population. The sense of coherence scale is commonly used in empirical research and has been used in at least 33 languages, with many different clinical populations [14]. This scale has been commonly used with family caregivers of people living with dementia but has not been validated in this population. Overall, good psychometric properties were demonstrated for using this scale in this population. In previous research, Cronbach’s α scores ranged from 0.70 to 0.92 across 127 studies [14] and good scores were also found within this psychometric evaluation (α = 0.88). Generally, the results from this psychometric evaluation reflected those reported in a previous systematic review of the reliability and validity of the sense of coherence scale in the general population [14]. Feasibility was also found to be high as there were no missing items or caregivers failing to complete the whole scale once they had started. This demonstrates that the scale was acceptable to caregivers in a self-report format. As the scale is normally used in an interviewer-administered format, it is promising to see that a self-report format did not affect completion rates. The scale had good internal consistency, with good Cronbach’s alpha scores for the entire scale and the subscales, which were similar for scores in the general population reported in a previous systematic review [14].

There were no floor and ceiling effects present in the scale as a whole or in any of the subscales within the population of family caregivers of people living with dementia. This indicates the suitability of the scale to be used in interventional research.

In terms of concurrent validity, all of the hypotheses were supported by the results from this study. Sense of coherence was associated with caregiver sense of competence and self-efficacy, which had not previously been studied in existing literature. Sense of coherence was strongly related to resilience, which was a unique finding with potentially important clinical implications. Previous literature has demonstrated an association between these variables but not to the same strength as this study [27]. However, this relationship warrants further investigation, given the strong association as it has not been well researched in caregivers of people living with dementia [28]. In addition, the correlation between sense of coherence to health-related quality of life demonstrates the importance of sense of coherence scale as a protective trait that may result in an improved health related quality of life for family caregivers, buffering against the challenges of being a caregiver. These findings reflect previous literature [11], which found that higher sense of coherence scores were associated with increased health related quality of life in caregivers.

The qualitative feedback demonstrated that caregivers felt that the items and scoring in the scale were not clear or needed updating as they were confusing, ambiguous, or offended the respondents, suggesting that the face validity could be improved. This may have arisen because the scale was developed for the general population.

### Methodological limitations of this research

Despite the large sample size, it proved difficult to engage family caregivers from more diverse ethnic groups, particularly non-white caregivers. The online survey also proved more popular than the paper one, indicating the importance of providing different response options to participants. This may have targeted a more specific population, which could explain the higher number of adult-child caregivers than most studies of a similar population. In order to address these issues, effort was made to engage with black and minority ethnic groups and charitable organisations, but this did not significantly increase the ethnic diversity of respondents. Additionally, over half of the caregivers were not cohabiting with the person with dementia, which may have impacted findings as it could indicate that they were either not the primary caregiver or that they represent a distinct group of caregivers who were more likely to engage with a survey. This may reduce the generalisability of the results. However, there were no statistically significant differences between caregivers who cohabited and those that did not on any of the constructs included in this study.

Additionally, the inclusion criteria remained broad in order to include a wide variety of caregivers in the sample in order for the sample to be self-selecting. However, this may limit the generalizability of the results to validate the instrument to a specific population however it may increase the utility of the instrument to be used with all types of caregivers.

We were not able to assess the content validity in detail due to the design of this survey. Therefore further investigation into the content validity of this scale is recommended with this population, both by consulting a panel of experts and by asking family caregivers in depth about their opinion of the items included in the scale.

### Future research

We recommend longitudinal or interventional research in order to investigate whether the SOC-13 is responsive to change and to evaluate test-re-test reliability in this population. Further guidance on potential modification should be considered, as responses from caregivers indicated some concerns about the face validity of the scale. Investigating this in a focus group would allow an in-depth examination into the face validity of the scale as a whole, and whether each item is suitable for use to measure sense of coherence in this population, leading to a potential revision of the scale.

In addition, findings from the CFA indicated that the factor structure of the SOC-13 was not confirmed to be a three-factor structure. Taken together with the qualitative findings, it indicates that the items and structure of the scale should be more comprehensively explored in terms of how well it measures sense of coherence, and the three subgroups within this domain (meaningfulness, comprehensibility and manageability). This could be explored in a focus groups design in order for caregivers to give an opinion as to how each of the questions reflect their own sense of coherence and measure how well they are able to find life meaningful, comprehensive and manageable.

## Conclusion

This was the first study to investigate the psychometric properties of this scale in this population and in a self-report format. Overall, the scale had good reliability and the psychometric properties were good, indicating that the Sense of Coherence scale-13 could be a suitable instrument for use in clinical practice and interventional research. Additional guidance for caregivers completing the scale could help to improve the face validity.

## Abbreviations

CFA: Confirmatory Factor Analysis
JDR: Join Dementia Research
RMSEA: Root Mean Square Error of Approximation
RS-14: Resilience Scale – 14 items
SEMD: Self- Efficacy for Managing Dementia Scale
SOC-13: Sense of Coherence Scale – 13 items
SPSS: Statistical Package for the Social Sciences
SRMR: Standardised Square Root Mean Residual
SSCQ: Short Sense of Competence Questionnaire
